# Reconsidering “Aging Well” According to Multiple Definitions: A Multidimensional Approach to Clinical Psychology of Aging

**DOI:** 10.3390/geriatrics9050120

**Published:** 2024-09-15

**Authors:** Luca Gaviano, Roberto Pili, Andrea Domenico Petretto, Roberta Berti, Gian Pietro Carrogu, Martina Pinna, Donatella R. Petretto

**Affiliations:** 1 Department of Pedagogy Psychology Philosophy, University of Cagliari, 09127 Cagliari, Italy; luca.gvn@gmail.com (L.G.); andreapetretto@yahoo.it (A.D.P.); roberta.berti80@libero.it (R.B.); gpcarrogu@gmail.com (G.P.C.); martina.pinna@hotmail.it (M.P.); 2 Worldwide Community of Longevity, 09032 Assemini, Italy; dott.robertopili@gmail.com

Aging is a phase of life that, though inevitable, includes an extraordinary variety of experiences, challenges, and opportunities. In the last decades, the growing interest for this subject led to a proliferation of studies and models aiming to understand what it means to age well. Aging was studied through several theoretical lenses, each one offering a unique perspective on the aging process; the perspective of the clinical psychology of ageing is one of these. The analysis of literature revealed a plurality of terms and definitions associated to aging, such as “successful aging”, “active aging”, “healthy aging”, “usual aging”, “positive aging” and many others. Such definitions are not just labels, but they reflect different philosophies and approaches guiding the research and politics on aging. Each of these terms tried to determine what it meant to age and most of all what it meant to live well and long [1]. Elsewhere, we discuss about those aging terms/definitions from the clinical psychology point of view providing ways to underline a clearer scheme of this complex phenomenon [2]; in this editorial we will briefly focus on some specific definitions.

The concept of “successful aging”, introduced by Rowe and Kahn in 1987, is one of the most influential and most used [3]. This model emphasizes three principal components: lack of illnesses and lack of disabilities, elevated cognitive and physical functioning, and active involvement in life. However, this definition was criticized for its normative approach, that may exclude people who, even though they face chronical diseases and disabilities, live a satisfactory and meaningful life [3]. For example, a study performed by Depp and Jeste found out that many elders with chronic disease may just the same reach a high level of satisfaction in life, questioning the rigidity of traditional definitions of successful aging [4].

In parallel, “active aging” promoted by World Health Organization (WHO) underlines the importance of continuous participation to social, cultural, economic, spiritual and civil [5]. This concept concentrates not only on physical and mental health, but also on emotional well-being and social integration. According to a report from WHO of 2002, active aging is based on the rights of elder people to achieve same opportunities and treatment in all spheres of life, reflecting a holistic and inclusive approach. Some other documents from WHO focused even more on those spheres of life [5]. 

The “positive aging” is another perspective stressing the capability of the individuals to adapt to changes and to keep an optimistic vision of life despite the challenges of age. This approach gives value to internal resources, like resilience and self-effectiveness, and external resources, like social support and access to services [6]. 

Ryff and Singer highlighted that psychological resilience and social support are fundamental for a positive aging, allowing the individuals to face better difficulties and to keep a high level of well-being [7].

Another model that is very spread and mentioned is the one proposed by Baltes and Baltes in 1990, the Model of Selection, optimization and compensation, also known the SOC model [8]. This model explores how the individuals face the challenges tied to aging and cognitive and functional deterioration [8]. Summarizing, the model offers a perspective to analyse and to understand how people adapt and face new challenges and losses. The objective of this model is not only to underline the losses, but also to highlight that, during aging, individuals may learn new skills and abilities. This is possible by using instruments, like technology or external resources coming, for example, from the social context in which the elder is inserted.

In parallel with the just mentioned approaches, also the medical traditional model dominated the research and medical practice for most of the 20th century, focusing mainly on biological aspects of disease and health. This model saw the human body as a machine, in which every malfunctioning could be diagnosed and treated with specific medical interventions. In the context of aging, this approach led to consider advanced age as an inevitable period of physical and mental decline, in which the main role of medicine was to manage chronical disease and associated symptoms [9]. However, with passing of time, the limitations of the medical model have become more and more evident. Scholars have started to recognize that health and well-being could not be explained completely in terms of physical pathology [1,10,11,12,13].

Another meaningful contribution is the evidence of the importance of a bio-psychological approach to aging, based on the approach for the first time by George Engel in 1977 [14]. Engel affirmed that to understand and treat illness, it was necessary to consider not only the biological factors, but the psychological and social one. In fact, the bio-psycho-social model suggests that biological factors, like genetics and physical health, interact with psychological factors, like aging perception, self-esteem and stress management, and social factors, like social support, community integration and participation opportunities. This integrated perspective offers a more complete and blurred understanding of aging, recognizing that individual experiences widely vary based on a series of interconnected influences. For example, studies have proven that the elders with a positive vision aging tend to live longer and in better health than the ones with a negative vision [15]. Besides, the social support was identified as a key factor in promoting the psychological well-being and resilience among elders [16]. The bio-Psycho-social model suggests then that to promote a healthy and successful aging, it is necessary to adopt an integrated approach keeping into account the interactions between biological, psychological and social factors. This meant that the intervention strategies must be multidimensional, including not only medical cures, but also psychological and social support [1,9]. 

This model recognizes that aging cannot be understood just through a biological optic, but it has to include psychological and social dimensions. Mental health, emotional well-being and social relationships are essential components for a healthy and successful aging [2]. 

The growing elderly population makes mandatory to implement interventions and programs aiming to promote the well-being during aging. Researchers underline that the need to explore and analyse the variables and determinants that allow an optimal aging process [17,18,19]. These interventions should be based on scientific evidence and keep into account the different dimensions of human experience. Programs of health promotions, psychological help initiatives and interventions of social inclusions are crucial to improve the quality of life of the elders. For example, adapted physical activities, support group, and continuous learning programs may help the elders to keep their autonomy and feel as an important part of community [20].

Researchers underline also the need to consider socio-cultural issues in the promotion of well-being during aging: the geographical and cultural diversity was of particular interest in the definitions of aging [21]. Elsewhere, we discussed on evidence and research coming from different regions of the world, highlighting how definitions and aging models vary meaningfully based on the cultural and social economic context [2]. This diversity offers a more complete and inclusive vision of the aging phenomenon, allowing to face the different perspective and practices in the different countries. It is noteworthy that in some cultures, aging is seen as a phase of wisdom and respect, while in other cultures it can be associated to a decline and marginalization [22]. For example, in Eastern societies, aging goes often together with an increase of respect and social position. Moreover, the elders are seen as keepers of knowledge and traditions, and their role is fundamental for keeping the family and community ties [23]. In contrast, in Western societies, aging can be perceived as a phase of decline and loss of autonomy, with a higher risk of social isolation and marginalization [24,25]. Those cultural differences have meaningful implications for projecting interventions and politics.

Because of great socio-cultural and cultural differences in the approaches on aging, a working approach in a cultural context may not work in another, highlighting the need for personalized solutions keeping into account cultural and social specificities, and lay perspective of ageing people [19]. For example, a study performed by Lamb [26] highlighted how in India, the elders living in extended families have a wider sense of belonging and support compared to elders in Western societies, in which the nuclear family structure prevails.

**Conclusions**: This editorial aims to offer an important contribution to understand aging from a psychological point of view. While the debate on definitions and aging modes remains open, a multi-dimensional and cultural approach is fundamental to fully understand this complex phenomenon [1,2,9,27]. The future of research on aging shall continue to explore such variables, promoting interventions to improve the quality of life of elder people in the whole world. In conclusion, reconsidering aging through a multi-dimensional psychological lens not only enriches our understanding of the process, but also offers new opportunities to promote well-being and quality of life in the elders. An approach integrating biological, psychological, and social aspects and recognizing the cultural and geographical diversity, is essential to develop inclusive and effective interventions. While the global population keeps on aging, it is fundamental that research and politics adapt to face the challenges and use in a positive way the opportunities of this important phase of life.
